# Reproductive Tract Tumours: The Scourge of Woman Reproduction Ails Indian Rhinoceroses

**DOI:** 10.1371/journal.pone.0092595

**Published:** 2014-03-26

**Authors:** Robert Hermes, Frank Göritz, Joseph Saragusty, Monica A. Stoops, Thomas B. Hildebrandt

**Affiliations:** 1 Department Reproduction Management, Leibniz Institute for Zoo and Wildlife Research, Berlin, Germany; 2 Center for Conservation and Research of Endangered Wildlife, Cincinnati Zoo and Botanical Garden, Cincinnati, Ohio, United States of America; Faculty of Animal Sciences and Food Engineering, University of São Paulo, Pirassununga, SP, Brazil, Brazil

## Abstract

In Indian rhinoceros, extensive leiomyoma, a benign smooth muscle tumour, was sporadically diagnosed post mortem and commonly thought of as contributing factor for reduced fecundity of this species in captivity. However, to date, the prevalence of reproductive tract tumours and their relevance for fecundity are unknown. Our analysis of the international studbook now reveals that females cease reproducing at the age of 18.1±1.2 years; equivalent to a reproductive lifespan of just 9.5±1.3 years. This short reproductive life is in sharp contrast to their longevity in captivity of over 40 years. Here we show, after examining 42% of the captive female population, that age-related genital tract tumours are highly prevalent in this endangered species. Growth and development of these tumours was found to be age-related, starting from the age of 10 years. All females older than 12 years had developed genital tumours, just 7–9 years past maturity. Tumour sizes ranged from 1.5–10 cm. With age, tumours became more numerous, sometimes merging into one large diffuse tumour mass. These tumours, primarily vaginal and cervical, presumably cause widespread young-age infertility by the age of 18 years. In few cases, tumour necrosis suggested possible malignancy of tumours. Possible consequences of such genital tract tumour infestation are hindered intromission, pain during mating, hampered sperm passage, risk of ascending infection during pregnancy, dystocia, or chronic vaginal bleeding. In humans, leiomyoma affect up to 80% of pre-menopause women. While a leading cause for infertility, pregnancy is known to reduce the risk of tumour development. However, different from human, surgical intervention is not a viable treatment option in rhinoceroses. Thus, in analogy to humans, we suggest early onset and seamless consecutive pregnancies to help reduce prevalence of this disease, better maintain a self-sustained captive population and improve animal welfare.

## Introduction

Four of the five extant rhinoceros species are listed by the International Union for Conservation of Nature and Natural Resources (IUCN) as being at risk of extinction. Poaching and illegal trade in rhinoceros horns are a leading factor in their demise. It was estimated that since the beginning of this year (2013), one rhino has been lost to poaching every 11 hours in Africa alone [1]. In Asia, the Javan (*Rhinoceros sondaicus*), Sumatran (*Dicerorhinus sumatrensis*), and Indian (*Rhinoceros unicornis*) rhinoceroses are also increasingly targeted for their highly profitable horns, worth US$ 150,000–200,000 a piece on the illegal market. Consequently, one of the last remaining Javan rhinoceros of mainland Asia has been poached in 2010 [2]. The Indian rhinoceros, once roaming throughout Southeast Asia, is now found only in two protected areas in India and Nepal and numbers around 2,900 animals [3], [4]. Because of the increasing anthropogenic pressure on these species *in situ*, a genetically diverse and proliferative *ex situ* population is of great importance. However, *ex situ* breeding is not really delivering the expected fruits.

The Indian rhinoceros was first introduced into Europe on 1^st^ May 1515. It was described shortly after in the text accompanying Albrecht Dürer famous wood carving print of the same year as “the wild beast that can even scare off an elephant”. Since this introduction, 439 years had passed before the first offspring was born in captivity in 1952 [5]. This poor performance may be because Indian rhinoceros long suffered from chronic foot disease in captivity and consequently, lack of breeding [6], [7]. Current husbandry guidelines aim to root this disease from the captive population [8]. However, despite husbandry changes, what remains is low fecundity or lack of reproduction in many captive females. The current genetic diversity of 90% is projected to further decline to 80.14% in the next 100 years [9]. To maintain genetic diversity of at least 90%, the introduction of up to 40 new founders is required [9]. Yet capturing new animals from the wild is not an option. Of the currently 189 animals of this rhinoceros species in captivity, distributed in over 70 zoological institutions, many produced no offspring. Furthermore, the genetic representation is significantly skewed towards a few overrepresented founders [10]. Almost 50% of all captive-born Indian rhinoceroses carry the genetic makeup of only three founders [11]. Stoops et al. [12] proposed that breeding and successful reproduction must occur with regularity between specifically paired animals in order to maintain a healthy and genetically diverse population. However, to date, none of the reports on reproduction biology in Indian rhinoceros addressed the reasons behind the fact that some females produce high number of offspring while others produce only few or none at all, despite their longevity in captivity [8]. A seemingly common explanation for reproductive failure in female Indian rhinoceroses is their assumed tendency to develop reproductive tract tumours, presumably leiomyoma. However, the evidence of leiomyoma in the Indian rhinoceros and its impact on fertility is scarce and merits further investigation. This is especially true since surgical removal of single tumours or complete hysterectomy, relatively simple surgical interventions in domestic species or humans are technically extremely challenging in rhinoceroses due to their thick integument and extended ribcage. Although laparoscopic biopsy has been reported in a white rhinoceros [13], extended surgical incisions impose high risk. The impeded surgical access for flank laparoscopy in rhinoceroses force us to seek other modalities, e.g. *in vivo* recovery of oocytes after ovarian superstimulation is performed transrectally [14]. Limited access to the animal for wound management further complicates seemingly simple surgical interventions in rhinos. It is therefore not surprising that the only reported attempt to perform hysterectomy in an Indian rhinoceros had a fatal outcome [15].

Leiomyomata are benign tumours of the smooth muscle tissue. They may occur anywhere in the body but probably the most common site is the female reproductive tract, primarily the uterus in humans and many other species, where they are sometimes referred to as uterine fibroids. In women, leiomyoma is the most common pelvic tumour and its incidence increases with age, reaching as much as 70–80% of the women by the age of 50 [16], [17]. These tumours start forming only after puberty and onset of the oestrous cycle activity, and in many cases they are associated with pain and increased menstrual bleeding [18]. Moreover, leiomyoma is a leading cause for infertility and is considered the number one indication for hysterectomy in women [17]. The risk of uterine leiomyoma is reduced in parous women by 40% and is further reduced in pluriparous women [19], [20]. Nevertheless, more than half of all affected women are likely to live throughout their lives without a diagnosis since leiomyoma often have no symptoms. Hormonal changes associated with menopause end further growth or formation of new tumours.

Leiomyoma is rarely reported in pets and livestock [21], possibly because of the common practice to perform ovariohysterectomy in young pets and because most livestock do not live beyond several years and are rarely diagnosed or treated as individuals. However, since the exact aetiology of these tumours is still not known, species-related differences may be involved here as well. Uterine leiomyoma has been sporadically diagnosed in a large number of wild terrestrial and marine mammals including gorilla, chimpanzee, spider monkey, macaque, leopard, cheetah, puma, rhinoceros, elephant, dolphin, and beluga whale [22]–[35]. However, due to lack of distinct symptoms, even excessive leiomyoma may remain undetected or be diagnosed incidentally, at far advanced age or, more commonly, post mortem [36]–[38]. The Asian elephant is the first wildlife species in which incidence of leiomyoma was described in a larger number of live animals [39]. Since then uterine leiomyoma is regarded as a leading cause for conception failure, early embryonic loss and endometritis in aged female Asian elephants (*Elephas maximus*) [36], [39], [40].

In rhinocerotidae, leiomyomata were reported in individual white (*Ceratotherium simum*), Sumatran, and Indian rhinoceroses [13], [31], [41]–[43]. In white rhinoceroses, leiomyoma has low prevalence compared to other reproductive tract lesions such as cystic endometrial hyperplasia or ovarian cysts [31]. In Indian and Sumatran rhinoceroses, leiomyoma is frequently mentioned in wildlife textbooks, husbandry guidelines, and numerous case reports [8], [44], yet a closer look reveals that the common impression concerning the prevalence of leiomyoma in Sumatran rhinoceros is based on the incidence of uterine masses found in just two females [45]. Similarly, the assumed prevalence in Indian rhinoceroses is based on post mortem reports of just nine females spanning over a period of more than 30 years [46].

To further understand the possible impact of reproductive tract tumours on captive reproduction in Indian rhinoceroses, we analysed the international studbook [5] to establish current female fecundity parameters in this population. Retrospective analysis of ultrasonographic examinations of almost half (42%) of the living female captive population was performed to establish the incidence and possible impact of reproductive tract tumours on the reproductive success in this species. We hypothesized that fecundity is associated with the incidence of reproductive tract tumours, presumably leiomyoma. If such relation is true, then the knowledge accumulated over years of research in human medicine may help in suggesting possible solutions for the captive rhino population. Based on our findings we compile recommendations that will, so we hope, facilitate higher fecundity to ensure maximum genetic diversity in the captive population of this endangered species. Through our approach to elucidate the incidence of reproductive tract tumours in the Indian rhinoceros, it is our aim to ignite research into other wildlife species *in situ* and *ex situ* in which reproductive tract tumours, that thus far have been diagnosed sporadically post mortem, may stand behind hitherto unexplained elevation in reproduction failure.

## Methods and Materials

### Ethics statement

This study was carried out in strict accordance with the German National Protection of Animals Act from 24.07.1972 and its last revision from 15^th^ July 2009. Under this Act, an examination directed towards diagnosing an animal's disease is not defined as an animal experiment (§7) but as a mandatory act of animal welfare. Under this Act (§5) anaesthesia is mandatory only if comparable procedures in humans require anaesthesia. Gynaecological examinations and associated diagnostic imaging as performed in this study do not require anaesthesia in humans, and technically do not require anaesthesia in rhinoceros either, if animals are properly conditioned. However, wild animals usually do not comply with veterinary diagnostic procedures and so, to achieve a level of tolerance to the otherwise non-harmful, painless gynaecological ultrasound examination, sedation or anaesthesia was performed in most females whose ultrasound data was used in this study. Although not mandated by law, the ultrasound examination protocol was still approved by the Leibniz Institute for Zoo and Wildlife Research Committee of Ethics and Animal Welfare and the Center for Conservation and Research of Endangered Wildlife, Cincinnati Zoo and Botanical Garden Institutional Animal Care and Use Committee (IACUC) (Permit Numbers: 1994-11-01 and 11-107, respectively).

### Studbook analysis

Data on date of birth, date of death, and dam of all female rhinoceroses was extracted from the 2011 International studbook [5]. Data on number of offspring, calving interval, and breeding output was calculated form individual data given for each female in the studbook. This was then used for a retrospective analysis of breeding output of the captive Indian rhinoceros population.

### Animals

Twenty-five female Indian rhinoceros (*rhinoceros unicornis*), 3 to 36 years of age, were assessed by ultrasonography for the incidence of reproductive tract tumours. Twenty-three females were still alive in 2011 when the international studbook of the Indian rhinoceros was compiled. They constitute over 42% of the mature and living female population. Seven of the females were reassessed after 8.3±1.8 years. The examinations were diagnostic interventions, aimed at clarifying the reproductive health of these females. The three year-old female was immature. All animals in this study are listed in the Indian rhinoceros International Studbook.

### Anaesthesia

Eight animals were trained to tolerate the examination without chemical restraint. In all other animals standing sedation (n = 10) or full anaesthesia (n = 7) was required [8], [47]. The choice of anaesthesia, sedation or voluntary examination was not standardized, as this choice had no impact on the data collected.

### Ultrasound examination

The reproductive organs were examined by transrectal ultrasonography as a standardized procedure in all captive rhinoceros species [12], [31]. Genital organs, including the vagina, cervix, uterus and ovaries, were imaged with a hand-held ultrasound probe in cross and longitudinal sections (Oculus CS 9100, 2–5 MHz probe, Hitachi, Physia GmbH, Neu-Isenburg, Germany; Voluson I, 2–5 MHz, GE Healthcare, Berlin, Germany; Sonosite Plus 180, Sonosite Titan, 4–2 MHz, SonoSite GmbH, Frankfurt a.M., Germany). Video sequences and still images of all ultrasound examinations were recorded for retrospective analysis.

### Statistical analysis

Statistical analysis was performed using GraphPad InStat (GraphPad Software Inc, Version 3.00, San Diego, CA, USA) and PASW Statistics for Windows v. 18.0.0 (SPSS Inc. Chicago, IL, USA) software. To compare means, unpaired two-tailed t-test was performed. Prior to analysis, the data passed the Kolmogorov and Smirnov normality test and distribution was considered normal when *P*>0.1. When standard deviations were not similar between populations, the unpaired two-tailed t-test was Welch corrected. All values are reported as mean ± SEM. For the purpose of this study, an ‘early breeder’ is defined as animal which became pregnant for the first time at or around the age of maturity (3 to 5 years [8]) and thus gave birth by the age of 7 years. A ‘late breeder’ is defined as animal that got pregnant past the age of maturity (>5 years). To characterize the relationship between age and incidence of genital tract tumours, a linear regression coefficient was calculated. Positive correlation coefficient was tested for its slope being significantly different from zero and its departure from linearity. Differences were considered significant when *P*<0.05.

## Results

### Studbook analysis

#### Fecundity of early and late breeders

The fecundity of early breeders was significantly higher than that of late breeders. In the non-living population of female Indian rhinoceros (n = 49), early breeders (1^st^ birth at 5.2±0.4 years, n = 9) gave birth to 6.6±1.2 calves during their lifetime, with per female offspring number ranging between 1–11 calves (Table 1). Despite a similar range of 1–12 calves born per female, late breeders (1^st^ birth: 12.5±0.8 years, n = 26) gave birth to 2.9±0.5 calves during their lifetime, a significantly smaller number (*P* = 0.0021). The balance 14/49 (28.6%) animals did not contribute offspring to the population at all. When all calves that were either stillborn or died within the first 3 months of their life were removed from this reproductive output, early breeders still produced more than twice as many offspring (5.2±0.1 calves) during their lifetime compared to late breeders (2.1±4.0). This difference in fecundity between early and late female breeders was highly significant (*P*<0.0001). While all early breeders contributed at least one surviving calf to the population, 19.2% (5/26) of the late breeders died without producing any live or surviving offspring. Late breeders without surviving offspring and females that never bred, both genetically not represented in the population, constituted 39% of the non-living female population. The studbook provided no further information that could explain the highly significant difference in fecundity between early and late breeders or the reason for the large proportion of non-breeders.

**Table 1:** Fecundity of early and late breeding female Indian rhinoceros in the non-living captive population.

Breeder category	Lifespan (y)	1st birth (y)	Last birth (y)	Intercalving interval (mo)	Last birth to death (y)	Calves born (#)	Calves dead (#)	Calves surviving (#)	Reprod. life (y)
Early breeder	22.2±3.1	5.2±0.4*	18.1±2.7	26.6±1.5*	4.1±2.7*	6.6±1.2*	1.3±0.6	5.2±1.0*	14.9±2.8*
Late breeder	27.7±2.0	12.5±0.8*	18.0±1.2	43.0±3.7*	9.7±1.3*	2.9±0.5*	0.8±0.2	2.1±0.4*	7.6±1.2*

* Values with asterisk within the same column were significantly different (*P*<0.05).

A similar significant difference in reproductive output between early and late breeders was calculated for the living population of female Indian rhinoceroses (n = 58, age range: 7–41 y). Again, early breeders (1^st^ birth: 5.5±0.3 y, n = 20) produced almost twice as many surviving offspring (2.9±0.4 calves) than late breeders (1^st^ birth: 11.3±0.6 years: producing 1.6±0.3 calves, n = 27) (Table 2). The balance 11/58 (19%) animals did not or not yet contribute to the population. Similar to results in the non-living population, the difference in fecundity between early and late female breeders in the living population was significant (*P*<0.019). Absolute calve numbers were lower than in the non-living population since remaining lifetime of these females give room for successive offspring output. It is worth noting that in the living population there are considerably more (*P* = 0.047) early breeders (20/56) compared to the dead population (9/49), indicating changes in management over the years.

**Table 2:** Fecundity of early and late breeding female Indian rhinoceros in the living captive population.

Breeder category	Age (y)	1st pregnancy (y)	1st birth (y)	Last birth (y)	Intercalving interval (mo)	Calves born (#)	Calves dead (#)	Calves surviving (#)
Early breeder	17.1±1.6	3.5±0.3*	5.5±0.3*	13.7±1.5	37.9±4.3	3.7±0.4*	0.8±0.2	2.9±0.4*
Late breeder	19.5±1.4	9.3±0.6*	11.3±0.6*	14.8±0.6	33.6±2.8	2.4±0.3*	0.9±0.3	1.6±0.3*

* Values with superscripts within the same column were significantly different.

#### Lifespan and reproductive lifespan

Whether a female was breeding or not had no significant influence on the animal's total lifespan. Non-breeders reached the age of 22.9±2.2 y, similar to proven breeders who reached the age of 26.3±1.8 y. The lifespan was, however, weakly correlated with the age at first calving. Females who delivered their first calf early in life had a longer lifespan compared to those giving first birth later in life or never (r = 0.3634, *P* = 0.0346). Breeding females stopped reproducing at the age of 18.1±1.2. Interestingly, when subdivided into early and late breeders, both stopped reproducing at the same age (18.1±2.7 years, range: 4–27 years; and 18.0±1.2 years, range 8–31 years, respectively). The age of reproductive cessation is significantly earlier compared with their total lifetime (*P*<0.05). No indications were found in the studbook as to why females, regardless of their reproductive output, would stop reproducing at such a young age.

Even though early and late breeders stopped reproducing at the same age, the period between the last birth and the death of a female was significantly shorter in early breeders (4.1±2.7 vs. 9.7±1.3 years, *P*<0.05). When calculating the reproductive life span of a female, here defined as the time between the first pregnancy and the last birth, early breeders began reproducing 7.3 years earlier and ceased breeding 5.6 years later than late breeders. The reproductive lifespan in early breeders was significantly longer, in fact twice as long as that of late breeders (14.9±2.8 vs. 7.6±1.2 years, *P*<0.0078). The reproductive lifespan was further found to be negatively correlated to the age at first calving. The later a female started reproducing the shorter her reproductive lifespan was (r = −0.4582, *P* = 0.0064).

#### Stillbirths and calf survival

Slightly more than half (51.2%, 42/82) of all reproducing females delivered at least one and up to five stillborn calves adding up to a total of 71 stillborn calves. This represents an overall stillborn rate in captivity of 25.9% (71/274). Of the females delivering a stillborn calf, 73.8% (31/42) were primiparous. In 40.5% (17/42) of these females, a stillborn calf was the last offspring they delivered. The studbook data fails to provide causes for the high stillborn rate and to explain why 40.5% of such females fail to become pregnant after delivering a stillborn. The rate of still born calves delivered by early and late breeders was not significantly different [0.2 (1.3/6.6) vs. 0.3 (0.8/2.9), *P*>0.05]. However, the number of surviving calves was negatively correlated to the females' age at first calving. The older a primiparous female was the lower the number of surviving offspring it had (r = −0.5250, *P* = 0.0014).

#### Intercalving interval

The overall intercalving interval of reproducing females in captive Indian rhinoceros was 35.8±1.8 months. When intercalving intervals were divided into those that followed a stillbirth and those that followed a live birth, intercalving interval following stillbirth was significantly shorter (28.9±2.4 vs. 37.8±2.4 months; *P*<0.0004). When intercalving interval of early and late breeders was compared, early breeders had a significantly shorter intercalving interval (26.6±1.5 vs. 43.0±3.7 months; *P* = 0.0032, table 1). In the living population the intercalving interval was not different between early and late breeders but significantly longer than in early breeders of the deceased population (37.9±4.3 vs. 33.6±2.8; *P* = 0.0233, *P* = 0.0374, respectively, table 2). The age at first calving was positively correlated with the length of intercalving interval in Indian rhinoceros. Thus the older a female was at fist calving, the longer was her intercalving interval throughout life (r = 0.5530, *P* = 0.0051). The combination of higher fecundity and shorter intercalving interval in early breeders results in a negative correlation between age at first calving and total number of calves born. The younger a female was at first calving, the higher number of calves she produced (r = −0.4761, *P* = 0.0044).

### Incidence of reproductive tract tumours

#### Ultrasound examination

Regular or irregular oestrous as indicator for ovarian function and prerequisite for breeding and conception was present in 85% (29/34) of the females during the course of the study (table 3). Females displaying anoestrous (4/5) were younger than 13 years and developed regular oestrous at a later stage of the study. Despite oestrous cycle activity in almost all females older than 13 years, eight females were mature but had remained nulliparous. Moreover, in 35.3% (6/17) of the proven breeders 4.7±1.2 years had passed since their last abortion, stillbirth or birth of a malformed calf, coinciding with data from the studbook where 40.5% of females failed to become pregnant again after stillbirth. In all females, no apparent causes for conception failure were noted prior to ultrasound examination.

**Table 3:** Reproductive history and ultrasound examination results.

Ultrasound	Studbook	Age	Level of	1st birth	Years since	Calves born	Abort,	Oestrous	Tumours	Max size	Fertility
assessments	(#)	(years)	restraint	(years)	last birth	(#)	stillborn (#)		(#)	(cm)	status
1	369	3	anaesthesia		never pregnant	0	0	anestrous	0		fertile
2	359	8	free standing		never pregnant	0	0	oestrous	0		fertile
3	367	9	free standing		never pregnant	0	0	anestrous	0		fertile
4	238	9	free standing		never pregnant	0	0	oestrous	0		fertile
5	269	9	sedation		pregnant	0	0	anestrous	0		fertile
6	193	10	anaesthesia		never pregnant	0	0	oestrous	0		fertile
7	256	10	sedation		pregnant	0	0	oestrous	0		fertile
8	274	10	sedation	4	4	2	0	oestrous	0		fertile
9	264	10	free standing	8	2	1	0	oestrous	0		fertile
10	185	12	anaesthesia	7	2	2	0	oestrous	0		fertile
11	144	12	anaesthesia		never pregnant	0	0	anestrous	0		fertile
12	189	13	free standing		never pregnant	0	0	oestrous	1	1,5	fertile
13	269	13	sedation	11	2	1	0	oestrous	2	1,50	fertile
14	245	13	sedation	12	1	1	1	oestrous	1	9,2	infertile
15	238	13	free standing		never pregnant	0	0	oestrous	3	3	fertile
16	230	14	free standing	5	2	2	0	oestrous	2	1,8	fertile
17	223	15	free standing	12	3	1	0	oestrous	2	1,5	fertile
18	97	16	anaesthesia		never pregnant	0	0	oestrous	10	5	infertile
19	241	16	sedation	8	4	2	0	oestrous	2	5	fertile
20	201	17	sedation	10	0	4	4	oestrous	3	1,7	fertile
21	256	17	sedation	11	3	2	0	anestrous	9	2,2	fertile
22	204	20	sedation	8	4	4	0	irr estrous	3	1,5	fertile
23	193	21	sedation	11	7	1	1	oestrous	18	2,6	fertile
24	189	21	free standing	17	2	2	2	oestrous	10	4,5	fertile
25	144	22	anaesthesia	20	2	1	1	oestrous	30	8	infertile
26	148	22	sedation	8	6	4	2	irr estrous	12	1,2	infertile
27	138	22	sedation	6	12	2	1	oestrous	8	7	infertile
28	67	23	anaesthesia		never pregnant	0	0	irr estrous	30	8	infertile
29	161	23	free standing	7	6	4	1	oestrous	17	6,6	infertile
30	193	24	sedation	15	1	1	1	oestrous	10	4,5	fertile
31	93	25	sedation	7	7	3	1	oestrous	21	6	infertile
32	93	32	sedation	7	7	3	1	oestrous	30	8	infertile
33	97	34	sedation		never pregnant	0	0	oestrous	15	10	infertile
34	40	36	anaesthesia	18	13	2	1	oestrous	30	9	infertile

irr oestrous  =  irregular oestrous.

**Figure 1:**
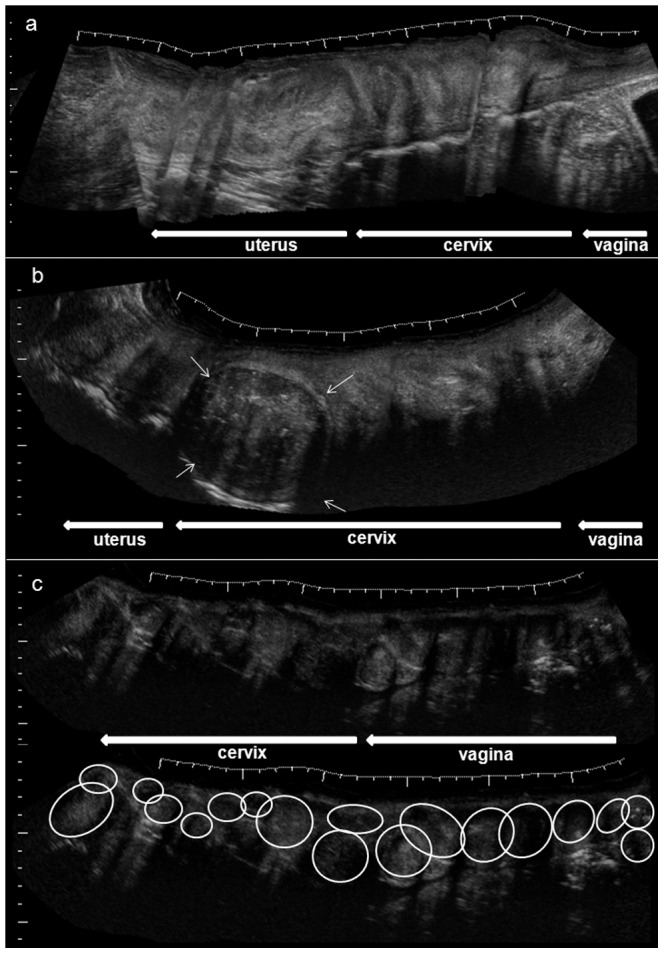
‘Extended field of view’ ultrasound images of the vagina, cervix, and uterus of Indian rhinoceroses with and without reproductive tract tumour. A. Extended sonogram of healthy vagina, cervix, and uterus. Beginning and end of the respective organs are indicated by the arrows underneath (←). B. Extended sonogram of vagina, cervix, and uterus with a single, large reproductive tract tumour present at the cranial aspect of the cervix (↓). C. Confluent, diffuse tumour present throughout the entire vagina and cervix. The organs wall and lumen are replaced by massive tumour overgrowth indicated by the circles.

**Figure 2:**
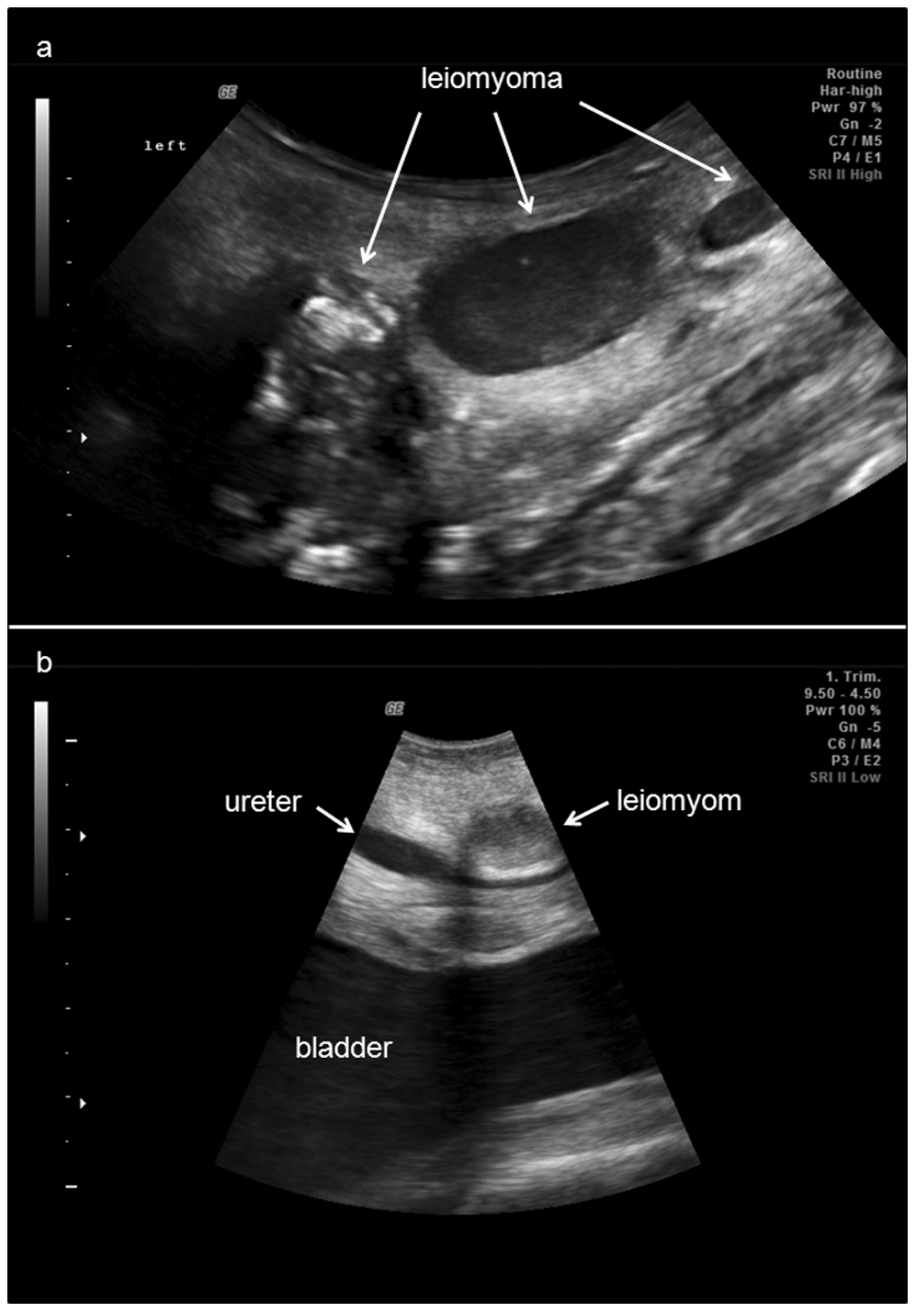
Ultrasound images of reproductive tract tumours in Indian rhinoceroses. A. Ultrasonographic appearance of reproductive tract tumours. Small and medium sized tumours appear as solid, dark, spherical structures. Large tumours may become necrotic indicated by high echoic centres. B. A vaginal tumour compresses the ureter above the bladder, causing partial obstruction and proximal dilation of the ureter.

Reproductive tumours found in the studied population were predominantly situated in the vagina and cervix (figure 1). In four females, uterine tumours were present in addition to neoplasm found in the vagina and cervix. Small tumours had low echogenic appearance. They presented as intramural or submucosal lesions with a diameter of 1.5 to 4 cm. The few large tumours (>4 cm) had a high echogenic centre, indicating tumour tissue necrosis (figure 2). In older females, tumour boundaries became diffuse, as tumours had merged, transforming into one large, diffused tumour mass (figure 1). In two cases, the vaginal tumour was in close proximity to the ureter, causing obstruction and retrograde dilation of the affected duct (figure 2). One of these females had become extremely aggressive, presumably due to advanced ureter obstruction and increased level of abdominal pain. Clinically observed bloody discharge (*n* = 1) was associated with large tumour masses surrounded by fluid filled cavities. These were regarded as the source of observed bloody discharge in this animal. None of the animals examined in this study, except the one with bloody vaginal discharge, had clinical symptoms prior to the ultrasound examination. Other lesions found were singular uterine cysts of 1–2 cm in two females.

The incidence of genital tract tumours detected by ultrasound in females older than 12 years of age was 100% (18/18, table 3). Tumour number and size per animal ranged between one and >30 and 1.5 to 10.0 cm, respectively. There was no difference in number or size of tumours between proven and non-proven breeders. Of the females diagnosed with neoplasm, 33.3% (6/18) were regarded as infertile due to excessive number or large size of the tumour masses. The youngest animal diagnosed as infertile was 13 years of age. Of those diagnosed as infertile, 33.3% (2/6) remained nulliparous and the balance 66.7% (4/9) were proven breeders but did not become pregnant again since. Even when the impact of smaller tumours on fertility remained speculative and females were still considered possibly fertile, only 27.8% (5/18) became pregnant again and gave birth to a live calf after tumour diagnosis. Thus, from a total of 18 females in which reproductive tumours were detected, 72.2% (13/18) did not produce offspring thereafter despite the presence of a regular oestrous cycle activity.

Both tumour number and maximum size correlated strongly with age (correlation coefficient for number of tumours: r = 0.8199, *P*<00001; for size: r = 0.5708, *P* = 0.0263). In all females in which ultrasound examination was repeated (after 8.3±1.8 years; *n* = 7), tumours had either newly developed or had increased in number and size, further demonstrating the progression of the disease with age (table 4). Grouping the 25 animals based on the presence of tumours resulted in two groups of 11 and 14 animals. Differences in age between the groups (9.27±0.73 y and 19.64±1.67 y, respectively) had a significant *P* value of 0.00002, indicating that the mean ages of the two groups appear to differ.

**Table 4:** Tumour incidence in female Indian rhinoceros – data from repeated ultrasound examinations.

Repeated	Studbook	Age	Tumours	Max tumour	Tumor	Fertility	Reproductive
ultrasound	(#)	(years)	(#)	diameter (cm)	necrosis	status	status
1	256	10	0			fertile	pregnant
	256	17	9	2,2	no	fertile	live calves
2	97	16	10	5	no	infertile	no pregnancy
	97	34	15	10	yes	infertile	no pregnancy
3	144	12	0			fertile	no pregnancy
	144	22	30	8	yes	infertile	no live calf
4	93	25	21	6	yes	infertile	live calves
	93	32	30	8	yes	infertile	live calves
5	189	13	1	1,5	no	fertile	no pregnancy
	189	21	10	4,5	no	fertile	no live calf
6	238	9	0			fertile	no pregnancy
	238	13	3	3	no	fertile	no pregnancy
7	193	10	0			fertile	no pregnancy
	193	21	18	2,6	no	fertile	no live calf

## Discussion

Analysis of the International Indian rhinoceros studbook revealed that regardless of the age at which females conceived for the first time, they all ceased reproducing at the age of ∼18 years. In other words, captive Indian rhinoceroses, on average, have a maximum reproductive lifespan of only about 14–15 years while their lifespan may sometimes exceed 35 or even 40 years. The fact that few individuals give birth up to the age of 30 years and beyond demonstrate that the reproductive lifespan of the Indian rhinoceros can potentially be ten or more years longer than the current average. No indications were found in the studbook to explain the discrepancy between the longevity of up to 46 years and the mostly low fecundity and short reproductive lifespan in captivity. Reproductive tumours found extensively in this study in all females older than 12 years, and absence of any other noteworthy genital pathology, present for the first time a plausible cause for this shortfall in fecundity in this species in captivity.

With an intercalving interval of about 27 months, early breeders produced on average 6.6 calves over their lifetime resulting in 5.2 live calves. This is more than twice as many calves when compared with fecundity of late breeders who produced only 2.1 live offspring. In the current living population intercalving interval is similar between the two groups of breeders, yet the number of surviving calves is still significantly higher in early breeders. However, the higher fecundity of early breeders is still far lower than the potential ideal fecundity of 9.3–10.0 calves during the same time frame of 14–15 years or 16.0 calves if an extended reproductive lifespan of 24 years, seen in few females in the population, is used. Although not documented for the Indian rhinoceros, such calculation assumes that animals could get pregnant during post-partum oestrous, thus creating an intercalving interval of 18 months, as was reported for other rhinoceros species [48]–[50]. Differences in lifetime offspring production are naturally in part due to the fact that early breeders had more years to reproduce, but it was also because apparently early breeders had shorter intercalving intervals. Had early breeders experienced the same intercalving interval as late breeders (43 months), they would have produced only 4.2 calves over their fertile years, rather than the 6.6 calves presently observed.

To provide proper breeding recommendations and improve genetic diversity in the future, a clear understanding of the causes for the suboptimal fecundity of Indian rhinoceros is required. In this study evaluating almost half of the current captive female population, we show that reproductive tract tumours are widely present in female Indian rhinoceroses. A 100% incidence of reproductive tract tumours in animals older than 12 y, infertility in 33.3% of tumour diseased females, lack of breeding success in 72.2% of females in which tumours were diagnosed, and the absence of other reproductive disorders, strongly suggest that reproductive tumours are a major contributing factor to the reduced fecundity in captive Indian rhinoceroses. We think that the extent of this reproductive disorder had been hugely underestimated in the past and is still largely unknown at present. Case reports gave a vague indication about the presences of leiomyomata in this species [43], [46], mainly based on findings in old deceased animals. The extent, dimension and location of leiomyoma in these earlier cases, in addition to four new confirmed leiomyoma cases (unpublished data; Zoological Garden Lisbon, personal communication; Zoological Society of London, personal communication), suggest that the reproductive tract tumours imaged in this study are, with high degree of probability, leiomyoma. Even though our study lacks histopathological proof (as in rhinoceroses this can safely be done only post mortem), the data presented here from live animals suggests, in analogy to previous pathology reports, that Indian rhinoceroses suffer from reproductive tract leiomyoma starting at a very early age. Just 7–9 years past maturity, at the age of 13 years, all animals studied showed reproduction-related problems and apparently had developed reproductive tract tumours that increased in size and number with age. Only five of the studied animals conceived or gave birth to a live calf after tumours had been diagnosed. Occasional tissue necrosis suggested potential malignancy of some of these tumours. Although tumour necrosis is also found in benign leiomyoma [51], a malignant character of some tumour masses is conceivable. To date all cases of reproductive tract tumours in Indian rhinoceros describe extensive but yet benign leiomyoma. Recent reports on metastasized, malignant uterine adenocarcinoma in one white, one Indian and one black rhinoceros [52], Fernandes personal communication; Bryant personal communication) suggest the presence of a variety of reproductive tumours in these species, some of which being malignant or even fatal.

In general, the similarity to humans is striking. In humans, leiomyoma, the most common reproductive tract tumour, start forming after puberty, at the onset of oestrous cycle activity. The strong correlation of reproductive tract tumours with age in the Indian rhinoceros seems to be almost a copy of the increased prevalence of leiomyoma with age in humans, where the cumulative prevalence by the time of menopause is 70–80% [16], [17], [53], [54]. However, unlike humans, where leiomyoma predominantly occurs in the uterus, tumours in Indian rhinoceroses were mostly situated in the vagina and cervix, acting as a physical barrier. Possible consequences of such a barrier include hindered intromission and pain during mating, hampered sperm passage through the cervix, risk of ascending infection during pregnancy, dystocia, and miscarriage. When tumours were located in the uterus, functional disturbance of the endometrium might impair successful implantation of the embryo and/or induce miscarriage. The high incidence of reproductive tract tumours and these possible consequences fit the breeding histories of the animals in this study: abortion, miscarriage, stillbirth, and, most importantly, conception failure despite regular oestrous cycles. All these complications are commonly observed with intramural leiomyoma in humans [55]–[57].

In regards to the mechanism and dynamics of growth of these tumours in rhinoceros, the vast knowledge from humans might shed some light. In women, tumour growth is dependent on the steroid hormones oestrogen and progesterone [58]. Although both hormones are usually regarded as tumour growth-promoting agents, they also cause growth restriction under certain circumstances. For instance, leiomyoma tumours rarely grow during pregnancy despite very high progesterone concentrations [59]. Pregnancy appears to also exert a certain protective effect, reducing the risk for developing leiomyoma [17], [58]. Multiparity further reduces this risk by 20 to 40% per parity. Thus humans with late menarche, becoming pregnant early in life and delivering multiple offspring have the lowest probability of developing leiomyoma or, if such have developed, its growth rate is considerably reduced. This protective effect of pregnancy or multiparity might also be present in rhinoceros, but is hard to measure. Still, in analogy to humans, it can be speculated that the longer reproductive lifespan in early breeders may, in part, be explained by similar possible protective effect exerted by parity in the Indian rhinoceros.

If we assume that reproductive tract tumours with consequent reproduction failure are inevitable and the age at which reproduction ceases is set due to tumour growth, then the closer to puberty the animal starts reproducing and the faster it conceives following each parturition, the larger the number of calves it can potentially contribute to the population. If, in addition, pregnancy and parity actually act as protecting factors, similar to humans, an early start and continuous breeding can be expected to help reducing the rate and severity of tumour growth and consequently extend the reproductive life beyond 18 years in captive Indian rhinoceroses. Late breeders in this study started reproducing at an age when the first tumours probably had already developed. Considering the threat of infertility from the age when these tumours can first occur and the average age of about 18 years at which females stop reproducing, the remaining time window in which late breeders are likely to produce offspring is roughly six years. In other words, they can be expected to produce, at most, two or three calves over their lifetime. Reproductive tract tumour development and subsequent shortfall in fecundity has therefore supposedly a tremendous impact on the genetic diversity of the captive population. About 19.2% of the late breeders and nulliparous females, constituting about 39% of the dead population, ended up not being genetically represented, as they fail to produce surviving offspring. More than 50% of all females give birth to a stillborn or a calf that does not survive the first three months of its life. The highest incidence of non-surviving offspring was found in primiparous females (73.8%), confirming previous calculations [11]. In addition, we found that for 20.7% of the females, a stillborn was the last offspring they produced. We speculate that the high incidence of reproductive tract tumours found in this study stand behind this observed reproductive problem. Undetected reproductive tract tumours in the birth canal of older females may complicate the birthing process to the extent of infant death during labour. Limited space in the pelvis and disrupted contractility due to extensive tumour presence may cause considerable birth complications. The extent of tumours, borderline during the last conception of these females, may not allow further conception thereafter.

Breeding success and captive population growth has in fact turned into a matter of concern, as the number of facilities suitable for housing Indian rhinoceros is limited. Addition of newborn animals to the current population is discussed as space challenge and regional programs have requested not to breed animals that are already represented in the population [60]. However, in light of our findings, recommendations to suspend breeding in females for any period of time may have tremendous impact on the individual's fecundity. If pregnancy has a delaying effect on reproductive tract tumour development in Indian rhinoceros as it does in humans, and considering the number and extent of tumours when the animals approach their average fertility failure age of about 18 years, recommendations to sustain breeding in this species may even become an animal welfare issue. Tumour necrosis or obstruction of the ureter or urethra as seen in advanced cases in this study may actually inflict incredible chronic pain. Such consequences can be minimized or even avoided if tumour growth is kept at bay by early start and continuous breeding efforts. As evident from the higher proportion of early breeders in the living population when compared to the dead population, changes in management over the years seem to be bearing fruits and should be encouraged. If, however, breeding has to be suspended, we suggest supplementing it with temporary down regulation of ovarian activity (Hermes *et al*. unpublished data), the driving force behind leiomyoma tumour development and progression.

In human medicine, one out of three patients with leiomyoma undergo surgery [17]. Single fibroids or the entire uterus are removed. With diffuse uterine leiomyomata, hysterectomy remains the only treatment. Other leiomyoma treatments such as myomectomy, radio frequency ablation, or tumour embolisation aimed at single tumours seem to improve fertility [17], [61]. Some of these surgical treatments might become optional interventions in rhinoceroses, but only in few and very selected cases. To date, the only reported attempt to perform hysterectomy in an Indian rhinoceros ended in fatal outcome [15]. Surgical treatment of single, large leiomyoma in young rhinoceros with a remaining breeding prospective in an attempt to re-install fertility represents a legitimate indication. However, when tumours become too numerous, surgery is not an option any longer. Treatment of females infested with diffused tumour growth should rather aim at improving animal welfare. Preliminary data shows that down regulation of hormonal activity halt further tumour growth in highly diseased females, thus improving their living conditions (Hermes *et al*. unpublished data).

In conclusion, high incidence of reproductive tract tumours and the finding that females are anticipated to become infertile by the age of about 18 years have tremendous impact on fecundity of female Indian rhinoceros in captivity. While longevity and clinical health in the absence of any disease symptoms appear not to be affected, fecundity seems to be greatly influenced by these tumours. If females are bred in proximity to puberty, fecundity can be increased by 148% or more when compared to late breeders. As long as the aetiology of these tumours remains speculative, ovarian activity starting at puberty, paired with lack of pregnancy, should be regarded as possible driving forces behind tumour growth and loss of fertility.

## References

[1] IUCN (2013) African rhinos won't hold out for much longer, IUCN experts warn. Available: http://www.webcitation.org/6IrjBYZBh. Accessed 2013 Aug 14.

[2] WWF (2010) Javan rhino found dead in Vietnam. Available: http://www.webcitation.org/6IrjW0nCz. Accessed 2013 Aug 14.

[3] Foose TJ, van Strien NJ (1997) Asian rhinos: Status survey and conservation action plan. Gland, Switzerland: IUCN. i-v, 1–112 p.

[4] Dinerstein E (2003) The Return of the Unicorns: The Natural History and Conservation of the Greater One-Horned Rhinoceros. New York: Columbia University Press. 384 p.

[5] von Houwald F, Pagan O (2012) Greater One-Horned or Indian Rhinoceros *Rhinoceros unicornis* Linné 1758, International Studbook 2011. Basel, Switzerland: Basel Zoo. 63 p.

[6] Galateanu G, Hildebrandt TB, Maillot A, Etienne P, Potier R, et al (2013) One small step for rhinos, one giant leap for wildlife management- imaging diagnosis of bone pathology in distal limb. PLoS ONE 8: e68493 10.1371/journal.pone.0068493 23874643 PMC3706412

[7] von Houwald FF (2001) Foot problems in Indian Rhinoceroses (*Rhinoceros unicornis*) in zoological gardens: Macroscopic and microscopic anatomy, pathology, and evaluation of the causes [Doctorate]. Zurich: Universität Zürich. 104 p.

[8] Guldenschuh G, von Houwald F (2002) Husbandry manual for the greater one-horned or Indian *rhinoceros unicornis* Linne, 1758. Basel, Switzerland: Basel Zoo. 94 p.

[9] Steck B (2010) Indian Rhino EEP; The European Association of Zoos and Aquaria Annual Conference, 22-25 September 2010; Verona, Italy. Available: http://www.eaza.net/News/verona2010/Pages/Proceedings.aspx.

[10] Hlavacek G, Zschokke S, Guldenschuh G (2002) International Studbook for the greater one-horned or Indian rhinoceros, *Rhinoceros unicornis*. Basel, Switzerland: Basel Zoo. 38 p.

[11] Zschokke S, Studer P, Baur B (1998) Past and future breeding of the Indian rhinoceros in captivity. Int Zoo News 45: 261–277.

[12] Stoops MA, Pairan RD, Roth TL (2004) Follicular, endocrine and behavioural dynamics of the Indian rhinoceros (*Rhinoceros unicornis*) oestrous cycle. Reproduction 128: 843–856 10.1530/rep.1.00328 15579602

[13] Radcliffe RM, Hendrickson DA, Lynn Richardson G, Zuba JR, Radcliffe RW (2000) Standing laparoscopic-guided uterine biopsy in a Southern white rhinoceros (*Ceratotherium simum simum*). J Zoo Wildl Med 31: 201–207 doi: 10.1638/1042-7260(2000)031[0201:SLGUBI]2.0.CO;2 10982133 10.1638/1042-7260(2000)031[0201:SLGUBI]2.0.CO;2

[14] Hermes R, Göritz F, Portas TJ, Bryant BR, Kelly JM, et al (2009) Ovarian superstimulation, transrectal ultrasound-guided oocyte recovery, and IVF in rhinoceros. Theriogenology 72: 959–968 10.1016/j.theriogenology.2009.06.014 19720394

[15] Klein LV, Cook RA, Calle PP, Raphael BL, Thomas P, et al. (1997) Etorphine-Isophlorine-O_2_ anesthesia for ovariohysterectomy in an Indian rhinoceros (*Rhinoceros unicornis*); Annual Conference of the American Association of Zoo Veterinarians, 26-30 October 1997; Houston, TX. pp. 127–130.

[16] Day Baird D, Dunson DB, Hill MC, Cousins D, Schectman JM (2003) High cumulative incidence of uterine leiomyoma in black and white women: Ultrasound evidence. Am J Obstet Gynecol 188: 100–107 10.1067/mob.2003.99 12548202

[17] Viswanathan M, Hartmann K, McKoy N, Stuart G, Rankins N, et al (2007) Management of uterine fibroids: An update of the evidence. Evid Rep Technol Assess 154: 1–122. PMC4781116 18288885

[18] Zimmermann A, Bernuit D, Gerlinger C, Schaefers M, Geppert K (2012) Prevalence, symptoms and management of uterine fibroids: an international internet-based survey of 21,746 women. BMC Women's Health 12: 6 10.1186/1472-6874-12-6 22448610 PMC3342149

[19] Parazzini F, La Vecchia C, Negri E, Cecchetti G, Fedele L (1988) Epidemiologic characteristics of women with uterine fibroids: A case-control study. Obstet Gynecol 72: 853–857. 3186092 10.1097/00006250-198812000-00008

[20] Parazzini F, Negri E, La Vecchia C, Chatenoud L, Ricci E, et al (1996) Reproductive factors and risk of uterine fibroids. Epidemiology 7: 440–442. 8793374 10.1097/00001648-199607000-00018

[21] King NW (1973) Comparative pathology of the uterus. In: Norris H, Hertig AT, Abell MR, editors. The Uterus. Baltimore, MD: The Williams and Wilkons Company. pp. 548.

[22] Mikaelian I, Labelle P, Doré M, Martineau D (2000) Fibroleiomyomas of the tubular genitalia in female beluga whales. J Vet Diagn Invest 12: 371–374 10.1177/104063870001200414 10907870

[23] Hernández-López L, Cerda-Molina AL, Páez-Ponce DL, Rojas-Maya S, Mondragón-Ceballos R (2007) Artificial insemination in black-handed spider monkey (*Ateles geoffroyi*). Theriogenology 67: 399–406 10.1016/j.theriogenology.2006.06.016 17023040

[24] Dejuste de Paula C, Neri Godoy S, Américo de Almida M, Kanamura C, Freitas Mota EF, et al. (2002) Leiomyoma in a captive puma (*Puma concolor*) in Brazil; Annual Conference of the American Association of Zoo Veterinarians, 5-10 October 2002; Milwaukee, WI. pp. 435–436 (abstract).

[25] Aballi Neninger OL (2002) Hysterosalpingographic and laparoscopic findings in an adult female chimpanzee (*Pan troglodytes troglodytes*); Annual Conference of the American Association of Zoo Veterinarians, 5-10 October 2002; Milwaukee, WI. pp. 428–430 (abstract).

[26] Young LA, Lung NP, Isaza R, Heard DJ (1996) Anemia associated with lead intoxiction and uterine leiomyoma in a chimpanzee (*Pan troglodytes*). J Zoo Wildl Med 27: 96–100.

[27] Burek KA, Mulcahy DM, Doroff AM, Comerci LR, Johnson TO (2000) Three sarcomas in freee-ranging Alaskan sea otters (*Enhydra lutris*); Annual Conference of the American Association of Zoo Veterinarians, 17-21 September 2000; New Orleans, LA. pp. 468 (abstract).

[28] Burns R (2006) Hemodialysis of a Western lowland gorilla (*Gorilla gorilla gorilla*) with fatal septicemia and pyelonephritis secondary to urine stasis and uterine leiomyosarcoma; Annual Conference of the American Association of Zoo Veterinarians, 19-24 September 2006; Tampa, FL. pp. 154–155 (abstract).

[29] Ford EW (1986) Obstetrical problems of nonhuman primates; Annual Conference of the American Association of Zoo Veterinarians, 2-6 November 1986; Chicago, IL. pp. 155–172.

[30] Gamble KC, North MCK, Backues K, Ross SR (2004) Pathologic review of the chimpanzee (*Pan troglodytes*): 1990-2003; Annual Conference of the American Association of Zoo Veterinarians, 28 August - 3 Aeptember 2004; San Diego, CA. pp. 561–566 (abstract).

[31] Hermes R, Hildebrandt TB, Walzer C, Göritz F, Patton ML, et al (2006) The effect of long non-reproductive periods on the genital health in captive female white rhinoceroses (*Ceratotherium simum simum*, C.s. cottoni). Theriogenology 65: 1492–1515 10.1016/j.theriogenology.2005.09.002 16213012

[32] Mylniczenko ND, Murrey SS, Smith S, Sewall LW, Facchini F (2008) Management of a uterine leiomyoma in a Western lowland gorilla (*Gorilla gorilla gorilla*); Joint conference of the American Association of Zoo Veterinarians and the Association of Reptile and Amphibian Veterinarians, 10–17 October, 2008; Los Angeles, California, USA. pp. 149 (Abstract).

[33] Owston MA, Ramsay EC, Rotstein D (2005) Incidence of neoplasia in a colony of captive felids at the Knoxville Zoological Park, 1979 to 2003; Annual Conference of the American Association of Zoo Veterinarians, 14–21 October 2005; Omaha, NE. pp. 100 (abstract).

[34] Pashov BN, Matamoros YH (1990) Veterinary observations about pacas (*Agouti paca*); Annual Conference of the American Association of Zoo Veterinarians, 21-26 October 1990; South Padre Island, TX. pp. 53–54 (abstract).

[35] Robert J (1986) Reproductive disorders in the male and female nonhuman primate: A brief overview; Annual Conference of the American Association of Zoo Veterinarians, 2-6 November 1986; Chicago, IL. pp. 173–180.

[36] Aupperle H, Reischauer A, Bach F, Hildebrandt T, Göritz F, et al (2008) Chronic endometritis in an Asian elephant (*Elephas maximus*). J Zoo Wildl Med 39: 107–110 10.1638/2006-0045.1 18432104

[37] Montali RJ, Hildebrandt T, Göritz F, Hermes R, Ippen R, et al.. (1997) Ultrasonography and pathology of genital tract leiomyomas in captive asian elephants: implications for reproductive soundness. Series Ultrasonography and pathology of genital tract leiomyomas in captive asian elephants: implications for reproductive soundness; Internationalen Symposiums über die Erkrankungen der Zootiere 38; Zurich, Switzerland. Akademie Verlag. pp. 253–258.

[38] Sapundzhiev E, Pupaki D, Zahariev P, Georgiev G, Ivanov I (2007) Fibroleiomyoma in elephant uterus. J Vet Med A 54: 499–500 10.1111/j.1439-0442.2007.00949.x 17931224

[39] Hildebrandt TB, Göritz F, Pratt NC, Brown JL, Montali RJ, et al (2000) Ultrasonography of the urogenital tract in elephants (*Loxodonta africana* and *Elephas maximus*): an important tool for assessing female reproductive function. Zoo Biol 19: 321–332 doi: 10.1002/1098-2361(2000)19:5<321::AID-ZOO4>3.0.CO;2-K

[40] Lueders I, Drews B, Niemuller C, Gray C, Rich P, et al (2010) Ultrasonographically documented early pregnancy loss in an Asian elephant (*Elephas maximus*). Reprod Fertil Dev 22: 1159–1165 10.1071/RD09305 20797354

[41] Hermes R, Hildebrandt TB, Göritz F (2004) Reproductive problems directly attributable to long-term captivity-asymmetric reproductive aging. Anim Reprod Sci 82–83: 49–60 10.1016/j.anireprosci.2004.05.015 15271443

[42] Schaffer NE, Agil M, Bosi E (2001) Utero - ovarian pathological complex of the Sumatran rhinoceros (*Dicerorhinus sumatrensis*). In: Schwammer HM, Foose TJ, Fouraker M, Olson D, editors; The International Elephant and Rhino Research Symposium, 7–11 June 2001; Vienna, Austria. Schülling Verlag. pp. 322.

[43] Montali RJ, Mann PC, Jones OM, Griner LA, Kuen GR, et al.. (1982) Leiomyomas in the genital tract of large zoo mammals. Series Leiomyomas in the genital tract of large zoo mammals; Internationalen Symposiums über die Erkrankungen der Zootiere 24, 19–23 May, 1982; Veszprém, Hungary. Akademie Verlag. pp. 117–122.

[44] Hermes R, Hildebrandt TB (2012) Rhinoceros theriogenology. In: Fowler ME, Miller RE, editors. Zoo and Wild Animal Medicine, Current Therapy. St. Louis, Missouri: Elsevier Saunders. pp. 546–561.

[45] Schaffer NE, Zainal-Zahari Z, Suri MSM, Jainudeen MR, Jeyendran RS (1994) Ultrasonography of the reproductive anatomy in the Sumatran rhinoceros (*Dicerorhinus sumatrensis*). J Zoo Wildl Med 25: 337–348.

[46] Göltenboth R (1995) Nashöner. In: Göltenboth R, Klös HG, editors. Krankheiten der Zoo- und Wildtiere. Berlin: Blackwell Wissenschaftsverlag. pp. 209–233 (German).

[47] Radcliffe RW, Morkel PV (2008) Rhinoceroses. In: West G, Heard D, Caulkett N, editors. Zoo Animal and Wildlife: Immobilization and Anesthesia. Oxford, UK: Blackwell Publishing Ltd. pp. 543–566.

[48] Jones ML (1977) De 'Zoological society of San Diego'. Zoo Anvers 42: 124–129.

[49] Rachlow JL, Berger J (1998) Reproduction and population density: trade-offs for the conservation of rhinos *in situ* . Anim Conserv 1: 101–106 10.1111/j.1469-1795.1998.tb00017.x

[50] Hildebrandt TB, Hermes R, Walzer C, Sos E, Molnar V, et al (2007) Artificial insemination in the anoestrous and the postpartum white rhinoceros using GnRH analogue to induce ovulation. Theriogenology 67: 1473–1484 10.1016/j.theriogenology.2007.03.005 17451805

[51] Hendrickson MR, Tavassoli FA, Kempson RL, McCluggage WG, Haller U, et al.. (2003) Mesenchymal tumours and related lesions. In: Tavassoli FA, P D, editors. WHO Classification of Tumours Pathology and Genetics: Tumours of the Breast and Female Genital Organs. 1st ed.Lyon, France: International Agency for Research on Cancer Press. pp. 233–244.

[52] Wilson M, Hermes R, Bainbridge J, Bassett H (2010) A case of metastatic uterine adenocarcinoma in a Southern white rhinoceros (*Ceratotherium simum simum*). J Zoo Wildl Med 41: 111–114 10.1638/2009-0128.1 20722262

[53] Walker WJ, Pelage JP (2002) Uterine artery embolisation for symptomatic fibroids: Clinical results in 400 women with imaging follow up. BJOG 109: 1262–1272 10.1046/j.1471-0528.2002.01449.x 12452465

[54] Cramer SF, Patel A (1990) The frequency of uterine leiomyomas. Am J Clin Pathol 94: 435–438. 2220671 10.1093/ajcp/94.4.435

[55] Sabry M, Al-Hendy A (2012) Medical treatment of uterine leiomyoma. Reprod Sci 19: 339–353 10.1177/1933719111432867 22378865 PMC3343067

[56] Cook H, Ezzati M, Segars JH, McCarthy K (2010) The impact of uterine leiomyomas on reproductive outcome. Minerva Ginecol 62: 225–236. 20595947 PMC4120295

[57] Goldberg J, Pereira L (2006) Pregnancy outcomes following treatment for fibroids: Uterine fibroid embolization versus laparoscopic myomectomy. Curr Opin Obstet Gynecol 18: 402–406 10.1097/01.gco.0000233934.13684.cb 16794420

[58] Okolo S (2008) Incidence, aetiology and epidemiology of uterine fibroids. Best Pract Res Clin Obstet Gynaecol 22: 571–588 10.1016/j.bpobgyn.2008.04.002 18534913

[59] Cooper NP, Okolo S (2005) Fibroids in pregnancy-Common but poorly understood. Obstet Gynecol Surv 60: 132–138. 15671902 10.1097/01.ogx.0000154688.02423.68

[60] Steck B (2012) Indian Rhino EEP; The European Association of Zoos and Aquaria Annual Conference, 26-29 September 2012; Innsbruck, Austria. On line only, Available: http://www.eaza.net/NEWS/INNSBRUCK2012/Pages/Proceedings.aspx.

[61] Kroon B, Johnson N, Chapman M, Yazdani A, Hart R, et al (2011) Fibroids in infertility – Consensus statement from ACCEPT (Australasian CREI Consensus Expert Panel on Trial vidence). Aust NZ J Obstet Gynaecol 51: 289–295 10.1111/j.1479-828X.2011.01300.x 21806566

